# Clinical impact of second radiotracer injection after failed lymphoscintigraphy in breast cancer sentinel lymph node biopsy: a retrospective cohort study

**DOI:** 10.1186/s12957-026-04513-1

**Published:** 2026-08-07

**Authors:** Leo Hänninen, Riitta I Aaltonen, Eeva Juhanoja, Anselm Tamminen

**Affiliations:** 1 Department of Clinical Medicine, Faculty of Medicine,, University of Turku, Turku, Finland; 2 Department of Plastic and General Surgery, Turku University Hospital, Kiinamyllynkatu 4-8, Turku, 20521 Finland; 3 Department of Oncology, Turku University Hospital, Turku, Finland

**Keywords:** Sentinel lymph node biopsy, Lymphoscintigraphy, Breast cancer, Non-visualization, Radiotracer injection, Subareolar, Axillary metastases

## Abstract

**Background:**

Sentinel lymph node biopsy (SLNB) is the standard method for axillary staging in breast cancer. To ensure the identification of sentinel lymph nodes (SLNs), lymphoscintigraphy (LS) is often used. However, failure of LS remains a clinical challenge. A second tracer injection has been shown to help improve the success of SLNB, but its association with surgical outcomes has been insufficiently studied. In this study, the effect of a second isotope injection on SLNB outcomes was investigated.

**Methods:**

This retrospective study included 2,466 patients undergoing LS with peritumoral radiotracer injection between 2010 and 2022. Second tracer injection was administered if initial LS was unsuccessful. Factors associated with failed initial LS were analyzed using univariable and multivariable methods. Propensity score matching (3:1) was performed to assess differences between patients with successful and failed initial LS. SLNB outcomes were evaluated.

**Results:**

Initial LS failed in 397 (16.1%) of patients. In multivariable analysis, older age, higher body mass index, tumor location and tumor multifocality were associated with LS failure. Tumors located in the upper medial quadrant were overrepresented among failed LS cases, whereas central tumors were underrepresented. SLNB was technically equally successful after failed and successful LS (97.5% in both groups). Patients with failed LS had a higher proportion of sentinel node-negative disease (OR 0.42, *p* < 0.0001).

**Conclusions:**

Second subareolar radiotracer injection after failed initial LS appears to be an effective salvage strategy for maintaining SLNB technical success. The observed lower nodal positivity rate among failed-LS patients may reflect altered lymphatic drainage patterns or patient selection and requires further validation.

## Introduction

 Sentinel lymph node biopsy (SLNB) is now the standard method for staging axillary lymph nodes in patients with early-stage breast cancer. Compared with axillary lymph node dissection (ALND), SLNB is associated with lower morbidity while providing equivalent staging [1, 2]. Various techniques using radioactive tracers have been developed to identify sentinel lymph nodes (SLNs), but no consensus exists regarding the optimal approach [3]. Typically, a radiocolloid tracer is injected prior to lymphoscintigraphy (LS) to visualize lymphatic drainage from the breast tumor and to localize SLNs before surgery.

Because lymphatic drainage patterns vary between individuals, this method enables patient-specific mapping of lymphatic vessels and identification of the nodes most likely to serve as initial metastatic targets [4].

Failure to visualize SLNs on LS is a recognized limitation in a subset of patients. This may be due to technical factors, such as inappropriate tracer particle size, incorrect imaging timing or injection technique [5, 6]. Notably, non-visualization occurs more frequently with intraparenchymal injections than with superficial methods [7]. In addition, patient-related factors including advanced age, elevated body mass index (BMI), and prior breast surgery can negatively affect visualization [8–10]. Furthermore, tumor involvement in the axilla may obstruct lymphatic flow, increasing the likelihood of non-visualization. Non-visualization, in turn, is associated with false-negative SLNB results [11, 12].

To address this issue, a second radiocolloid tracer injection has been proposed to improve SLN detection and reduce SLNB failure rates [13]. It has been previously demonstrated that, in cases of axillary non-visualization, a second tracer injection significantly improved SLN identification and overall SLNB success rates, thereby reducing the need for unnecessary ALND in patients without axillary disease [14]. However, there is currently limited evidence as to whether SLNs identified after a second injection are equivalent to those identified after the initial injection [15].

The aim of this study was to evaluate, in a single-center patient cohort, how SLNs identified after a second radiocolloid tracer injection differ from those identified after the initial injection in terms of the number of removed and positive SLNs.

## Methods

### Study population

This study included patients who underwent primary breast cancer surgery with SLNB using peritumoral isotope injection for invasive breast cancer at Turku University Hospital between 2010 and 2022. All patients underwent preoperative axillary ultrasound and were cN0. If axillary metastases were detected preoperatively, patients underwent axillary lymph node dissection (ALND). Patient information was retrieved from the Auria Clinical Informatics Register.

The research protocol of the study was approved by the Hospital District of Southwest Finland (T537/2022).

### Sentinel node biopsy protocol

For SLNB, a radiotracer was injected into breast tissue surrounding the tumor. When tumors were palpable, injections may have been administered under palpation guidance, whereas ultrasound or stereotactic guidance was used for non-palpable lesions. In each case, a radiologist specializing in breast imaging performed the injection. After administration, sufficient time (approximately 2 h), was allowed for the tracer to enter lymphatic vessels and migrate toward regional lymph nodes. LS was performed in each patient. The anatomical location of visualized SLNs was marked on the skin prior to surgery.

In situations where SLNs were not visualized on initial imaging, an additional subareolar tracer injection was administered to improve detection, provided that it did not compromise localization of the primary tumor based on the initial injection. Repeat imaging was performed, when scheduling permitted, to confirm SLN localization. According to institutional records, 5–8% of patients managed with a single injection had a failed initial LS but did not undergo reinjection. However, these patients could not be identified individually within the study dataset.

LS examinations were not reanalyzed for this study. Instead, the study relied on the original clinical assessments recorded during routine patient care. Failed LS was defined as the absence of radiotracer accumulation considered compatible with a SLN in the ipsilateral axilla. Internal mammary and other extra-axillary drainage patterns were recorded; however, examinations without axillary visualization were classified as failed, as the primary objective of LS in this setting was the identification of axillary SLNs.

At minimum five minutes prior to surgery, a blue dye tracer was injected subareolarly to facilitate intraoperative identification of SLNs. During surgery, SLNs were located using both a gamma probe detecting radioactivity and visual inspection for blue-stained lymphatic vessels and nodes. All lymph nodes demonstrating focal radioactivity or blue staining were excised. After removal of identified SLNs, the axilla was palpated and any suspicious lymph nodes were excised for histopathological analysis. SLNB was regarded as successful only when at least one radioactive or dye-stained lymph node was identified and removed. If no SLN could be detected intraoperatively, ALND was performed. 

### Data collection

The following variables were collected: age, BMI, BMI category, ASA classification, tumor quadrant location, molecular subtype of the cancer, tumor diameter, TNM T-stage, result of the first LS, tumor laterality, type of breast surgery performed, lymph node surgery performed, presence of invasion and/or DCIS, multifocality, hormone receptor and HER2 status, tumor proliferation rate, histological grade, presence of SLN and overall lymph node metastases, number of examined sentinel and total lymph nodes, and TNM N-stage. 

### Statistical analysis

The data were analyzed using JMP 19 Student Edition (SAS Institute, Cary, North Carolina, USA) analysis software. To evaluate risk factors for failed LS, statistical analysis was performed. A two-sample t-test was performed for normally distributed variables, Wilcoxon test for non-normally distributed continuous variables and a chi-square test was used for categorical variables. Two-sided p-values < 0.05 were considered statistically significant. All variables having a relationship *p* < 0.15 were included in the multivariable logistic regression analysis. While performing the logistic regression analysis the variables with the highest p-value were eliminated one by one until only factors with statistical significance (*p* < 0.05) were remaining. Variables that remained statistically significant in the multivariable model were subsequently included in propensity score matching. As the primary objective was to evaluate the impact of LS on SLNB success, propensity score matching was used to create balanced groups for the final analysis, with risk factors for failed LS taken into account during the balancing process. Since there were significantly fewer participants whose first LS failed compared to the ones with successful LS, the matching was performed with a 3:1 ratio. Propensity score matching was performed using the nearest neighbor method with a caliper of 0.02.

Covariate balance between the matched groups was assessed using absolute standardized mean differences (SMDs), with values < 0.10 considered indicative of good balance and values < 0.20 considered acceptable.

## Results

**Fig. 1:**
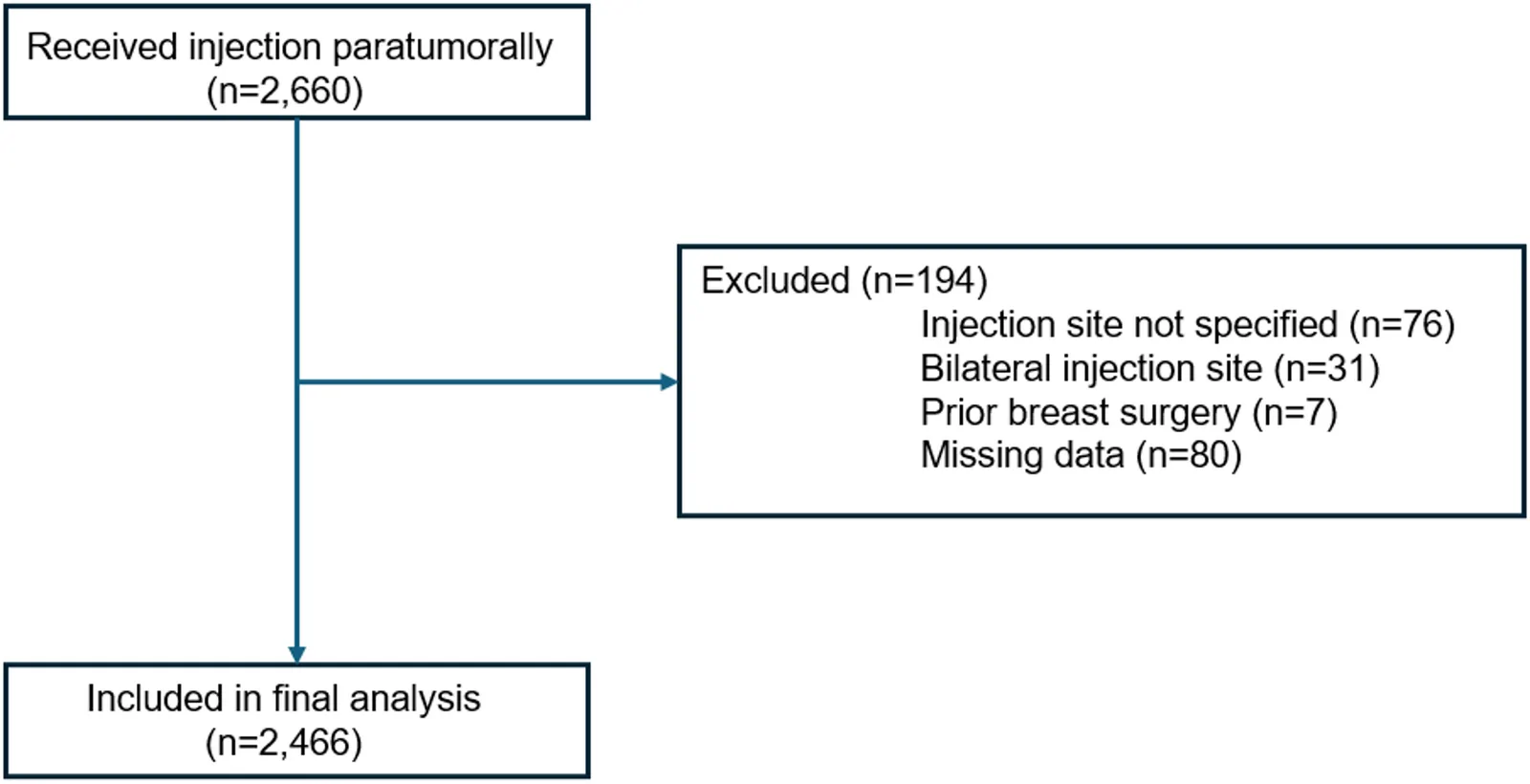
Patient selection for the study

Initially, 2,660 patients who had received a peritumoral injection for LS were identified. After exclusions, 2,466 patients met the eligibility criteria and were included in the final analysis (Fig. 1).

Baseline patient and tumor characteristics are presented in Table 1.

**Table 1:** Patients and characteristics. Values are *n* (%) unless specified otherwise. (IQR = interquartile range, BMI = body mass index, ASA = American Society of Anesthesiologists, HR = hormone receptor, HER2 = human epidermal growth factor receptor 2)

Age (years), median (IQR)	*n* = 2466
< 50	242 (9.8)
50–69	1668 (67.6)
70+	556 (22.6)
BMI (kg/m^2^), median (IQR)	27.0 (23.9–31.2)
ASA classification	
ASA 1	436 (17.7)
ASA 2	1406 (57.1)
ASA 3	593 (24.1)
ASA 4	26 (1.1)
No data	5
Tumor Quadrant	
Upper medial	479 (19.4)
Upper lateral	1417 (57.5)
Central	97 (3.9)
Lower medial	177 (7.2)
Lower lateral	296 (12.0)
Cancer type	
Ductal	2059 (83.5)
Lobular	269 (10.9)
Other	138 (5.6)
Cancer tumor diameter (mm), median (IQR)	14 (10–20)
TNM-T stage	
T1a	90 (3.6)
T1b	629 (25.5)
T1c	1157 (47.0)
T2	563 (22.8)
T3	25 (1.0)
Multifocality	
Yes	417 (16.9)
No	2049 (83.1)
HR	
Positive	2321 (94.5)
Negative	136 (5.5)
No data	9
HER2	
Positive	185 (7.5)
Negative	2272 (92.5)
No data	9
Proliferation index Ki-67 (%), median (IQR)	15 (8–25)
Grade	
1	566 (24.3)
2	1236 (53.1)
3	527 (22.6)
No data	137

The LS failed and a second injection was administered subareolarly to 397 (16.1%) out of the 2466 patients. The data were divided into successful and failed first LS groups. Relative percentages were calculated according to the success of the first injection to facilitate comparison between groups (Table 2).

**Table 2:** Comparison of patients with successful and failed lymphoscintigraphy. Values are *n* (%) unless specified otherwise. (LS = lymphoscintigraphy, IQR = interquartile range, BMI = body mass index, ASA = American Society of Anesthesiologists, HR = hormone receptor, HER2 = human epidermal growth factor receptor 2, NS = not significant, SMD = standardized mean difference)

Total (2466)	Successful LS	Failed LS, second injection administered	Absolute SMD	Univariable analysis	Multivariable analysis + odds ratio, CI (95%)
Age (years)				*p* = 0.0188	*p* = 0.0140
< 50	216 (10.4)	26 (6.6)	0.14		Reference
50–69	1401 (67.7)	267 (67.3)	0.01		1.61 (1.04–2.47)
70+	452 (21.9)	104 (26.2)	0.10		1.93 (1.21–3.07)
BMI category (kg/m^2^)				*p* = 0.0061	NS*
15–20	50 (3.9)	6 (2.2)	0.10		
20–25	400 (31.1)	62 (29.7)	0.18		
25–30	467 (36.3)	98 (36.3)	0.00		
30–35	243 (18.9)	60 (22.2)	0.08		
35–40	88 (6.8)	30 (11.1)	0.15		
> 40	38 (3.0)	14 (5.2)	0.11		
ASA classification				*p* < 0.0001	NS*
ASA 1	398 (19.3)	38 (9.6)	0.28		
ASA 2	1180 (57.1)	226 (57.1)	0.00		
ASA 3	465 (22.5)	128 (32.3)	0.22		
ASA 4	22 (1.1)	4 (1.0)	0.01		
Tumor Quadrant				*p* = 0.0004	*p* = 0.0002
Lower lateral	252 (12.2)	44 (11.1)	0.03		Reference
Lower medial	151 (7.3)	26 (6.5)	0.03		0.96 (0.57–1.63)
Central	91 (4.4)	6 (1.5)	0.17		0.38 (0.16–0.93)
Upper lateral	1200 (58.0)	217 (54.7)	0.07		1.04 (0.73–1.48)
Upper medial	375 (18.1)	104 (26.2)	0.20		1.63 (1.11–2.41)
Cancer type				*p* = 0.705	
Ductal	1723 (83.3)	336 (84.6)	0.04		
Lobular	227 (11.0)	42 (10.6)	0.01		
Other	119 (5.7)	19 (4.8)	0.04		
TNM-T stage				*p* = 0.5750	
T1a	75 (3.6)	15 (3.8)	0.01		
T1b	513 (24.8)	116 (29.3)	0.10		
T1c	976 (47.2)	181 (45.7)	0.03		
T2	483 (23.4)	80 (20.2)	0.08		
T3	21 (1.0)	4 (1.0)	0.00		
Multifocality				*p* = 0.0013	*p* = 0.0468
No	1698 (82.1)	351 (88.4)	0.18		1.65 (1.19–2.30)
Yes	371 (17.9)	46 (11.6)	0.18		Reference
HR				*p* = 0.8348	
Negative	115 (5.6)	21 (5.3)	0.01		
Positive	1947 (94.4)	374 (94.7)	0.01		
HER2				*p* = 0.3834	
Negative	1911 (92.7)	361 (91.4)	0.05		
Positive	151 (7.3)	34 (8.6)	0.05		
Proliferation				*p* = 0.8659	
< 20	1215 (59.0)	235 (59.5)	0.01		
≥ 20	843 (40.1)	160 (40.5)	0.01		
Grade				*p* = 0.7311	
1	473 (24.2)	93 (24.7)	0.01		
2	1043 (53.4)	193 (51.3)	0.04		
3	437 (22.4)	90 (24.0)	0.04		

In univariable analysis several factors were associated with non-visualization after the first injection, including age (*p* = 0.0188), BMI (*p* = 0.0061), ASA-class (*p* < 0.0001), tumor location quadrant (*p* = 0.0004) and tumor multifocality (*p* = 0.0013). Patients with failed LS were older and more frequently belonged to higher BMI categories.

In multivariable analysis the patients who had been given a second injection subareolarly were older than the ones who received only one injection (70 + groups 21.9% vs. 26.2%, *p* = 0.0140). The quadrant location of the breast tumor was also significant (*p* = 0.0002), and the upper medial quadrant was more frequently observed in patients with failed LS (26.2% vs. 18.1%), whereas the central tumors were underrepresented in this group (1.5% vs. 4.4%). In addition, multifocal tumors were more likely to have success in LS imaging (17.9% vs. 11.6%, *p* = 0.0468).

The relationship between LS success and axillary surgery outcomes is shown in Table 3.

**Table 3:** Sentinel lymph node biopsy outcomes in entire patient cohort. Values are *n* (%) unless specified otherwise. (LS = lymphoscintigraphy, IQR = interquartile range, SLN = sentinel lymph nodes, SLNB = sentinel lymph node biopsy, ALND = axillary lymph node dissection)

	Total *n* = 2466	LS successful	LS failed, second injection administered	Univariable analysis
SLNB outcome				*p* < 0.0001
SLNB with negative lymph nodes	1945 (78.9)	1599 (77.3)	346 (87.2)	
Positive lymph nodes and subsequent ALND	460 (18.7)	419 (20.2)	41 (10.3)	
Unsuccessful SLNB and subsequent ALND	61 (2.5)	51 (2.5)	10 (2.5)	
Number of metastases (SLN)				*p* < 0.0001
0	1849 (77.6)	1518 (75.8)	332 (87.1)	
1	414 (17.3)	381 (19.0)	33 (8.7)	
2	97 (4.1)	84 (4.2)	13 (3.4)	
3	20 (0.1)	19 (1.0)	1 (0.3)	
4	2 (0.04)	0 (0)	2 (0.5)	
Examined axillary sentinel lymph nodes, mean (SD)		1.87 (0.93)	2.00 (1.00)	*p* = 0.0211
pN-stage (ALND data included)				*p* < 0.0001
pN0	1887 (76.5)	1551 (74.9)	336 (84.6)	
pN1	514 (20.8)	463 (22.4)	51 (12.9)	
pN2	51 (2.1)	45 (2.2)	6 (1.5)	
pN3	14 (0.6)	10 (0.5)	4 (1.0)	

A successful SLNB is defined as the removal of at least one radioactive or dye-stained lymph node. Interestingly, failure of LS and administration of a second tracer injection did not affect the success of SLNB, as the procedure failed in 2.5% of cases in both groups. However, slightly more SLNs were removed in the second tracer injection group (2.00 vs. 1.87, *p* = 0.021), a small absolute difference of limited clinical relevance, and no longer statistically significant in the matched cohort, while the proportion of pN+ patients in this group was significantly lower (15.4% vs. 25.1%, *p* < 0.0001).

Propensity score matching was performed to improve comparability between the study groups. Variables included in the propensity score-matching model were selected based on their identification as independent predictors of failed LS in the multivariable analysis and are presented in Table 2. Because the number of patients with failed initial LS who underwent a second injection was substantially smaller than that of patients with initially successful LS, 3:1 propensity score matching was used. The final matched cohort included 1,040 patients (780 with initially successful LS and 260 with failed initial LS and second injection administered), while 86 patients from the failed-LS group were excluded during the matching process. As seen in Table 4, propensity score adjustment effectively reduced baseline differences between the groups.

**Table 4:** Comparison of baseline characteristics in propensity matched cohorts. Values are *n* (%) unless specified otherwise. (LS = lymphoscintigraphy, IQR = interquartile range, BMI = body mass index, ASA = American Society of Anesthesiologists, HR = hormone receptor, HER2 = human epidermal growth factor receptor 2, SLNB = sentinel lymph node biopsy, ALND = axillary lymph node dissection, SMD = standardized mean difference)

	Total (1040)	LS successful	LS failed, second injection administered	Absolute SMD	Univariable analysis
Age category (years)					*p* = 0.2863
< 50	57 (5.5)	41 (5.3)	16 (6.2)	0.04	
50–69	688 (66.2)	508 (65.1)	180 (69.2)	0.09	
70+	295 (28.4)	231 (29.6)	64 (24.6)	0.11	
Surgery (breast)	1040				*p* = 0.0657
Breast-conserving Surgery	962 (92.5)	715 (91.7)	247 (95.0)	0.13	
Mastectomy	78 (7.5)	65 (8.3)	13 (5.0)	0.13	
BMI categories (kg/m^2^)					*p* = 0.9175
15–20	27 (2.6)	21 (2.7)	6 (2.3)	0.02	
20–25	266 (25.6)	204 (26.2)	62 (23.8)	0.05	
25–30	372 (35.8)	275 (35.3)	97 (37.3)	0.04	
30–35	233 (22.4)	174 (22.3)	59 (22.7)	0.01	
35–40	103 (9.9)	75 (9.6)	28 (10.8)	0.04	
> 40	39 (3.7)	31 (4.0)	8 (3.1)	0.05	
ASA classification					*p* = 0.0784
ASA 1	123 (11.9)	102 (13.1)	21 (8.1)	0.16	
ASA 2	588 (56.7)	430 (55.3)	158 (61.0)	0.12	
ASA 3	311 (30.0)	233 (29.9)	78 (30.1)	0.00	
ASA 4	15 (1.4)	13 (1.7)	2 (0.8)	0.08	
Quadrant					*p* = 0.9792
Lower lateral	131 (12.6)	99 (12.7)	32 (12.3)	0.01	
Lower medial	71 (6.8)	52 (6.7)	19 (7.3)	0.03	
Central	18 (1.7)	14 (1.8)	4 (1.5)	0.02	
Upper lateral	555 (53.4)	419 (53.7)	136 (52.3)	0.03	
Upper medial	265 (25.5)	196 (25.1)	69 (26.6)	0.03	
Cancer type					*p* = 0.9712
Ductal	881 (84.7)	660 (84.6)	221 (85.0)	0.01	
Lobular	112 (10.8)	85 (10.9)	27 (10.4)	0.02	
Other	47 (4.5)	35 (4.5)	12 (4.6)	0.01	
TNM-T stage					*p* = 0.2095
T1a	37 (3.5)	25 (3.2)	12 (4.6)	0.07	
T1b	276 (26.5)	197 (25.2)	79 (30.4)	0.11	
T1c	477 (45.7)	357 (45.8)	120 (46.1)	0.01	
T2	237 (22.8)	191 (24.5)	46 (17.7)	0.17	
T3	13 (1.3)	10 (1.3)	3 (1.2)	0.01	
Multifocality					*p* = 0.1060
No	867 (83.4)	642 (82.3)	225 (86.5)	0.12	
Yes	173 (16.6)	138 (17.7)	35 (13.5)	0.12	
HR					*p* = 0.8873
positive	969 (93.6)	727 (93.7)	242 (93.4)	0.01	
negative	66 (6.4)	49 (6.3)	17 (6.6)	0.01	
HER2					*p* = 0.5052
positive	78 (7.5)	56 (7.2)	22 (8.5)	0.05	
negative	957 (92.5)	720 (92.8)	237 (91.5)	0.06	
Proliferation (%), mean (IQR)	14 (8–25)	14 (8–25)	15 (7–25)	0.04	*p* = 0.8342
Grade					*p* = 0.2520
1	234 (24.0)	174 (23.9)	60 (24.2)	0.01	
2	525 (53.8)	401 (55.1)	124 (50.0)	0.10	
3	217 (22.2)	153 (21.0)	64 (25.8)	0.11	

Covariate balance after propensity score matching was assessed using SMDs. The matched groups demonstrated good balance across most measured covariates, with the majority of SMD values below 0.10 and all values below 0.20, suggesting adequate comparability with respect to the observed characteristics.

In the propensity score–matched cohort as well, the group of patients with failed LS who received a second injection had a clearly higher proportion of pN0-patients compared to the group in which LS was successful after the first injection. The difference in the number of examined SLNs was essentially the same as in the overall cohort; however, due to the smaller sample size, the p-value was not statistically significant.

SLNB results were again compared between these matched groups, and the following results were obtained (Table 5).

**Table 5:** Axillary surgery outcomes in propensity matched cohorts. Values are *n* (%) unless specified otherwise. (LS = lymphoscintigraphy, SLNB = sentinel lymph node biopsy)

	Total (1040)	LS successful (780)	LS failed, second injection administered (260)	Univariable analysis
SLNB unsuccessful	27 (2.7)	22 (2.8)	5 (1.9)	*p* = 0.43
Examined sentinel lymph nodes (mean, 95% CI)		1.87 (1.78–1.96)	1.99 (1.84–2.14)	*p* = 0.15
Metastatic sentinel lymph nodes				*p* < 0.0001
0	786 (77.8)	562 (74.1)	224 (87.8)	
1	173 (17.0)	152 (20.0)	21 (8.3)	
2	45 (4.4)	37 (4.9)	8 (3.1)	
3	8 (0.8)	7 (0.9)	1 (0.4)	
4	1 (0.1)	0 (0)	1 (0.4)	
Information missing	1	0	1	
pN stage				*p* < 0.0001
N0	799 (76.8)	573 (73.5)	226 (86.9)	
N1	214 (20.6)	185 (23.7)	29 (11.2)	
N2	22 (2.1)	19 (2.4)	3 (1.1)	
N3	5 (0.5)	3 (0.4)	2 (0.8)	

When risk factors for axillary-positive disease were analyzed in the propensity score–matched cohort, the following results were obtained (Table 6).

**Table 6:** Risk factors for pN+ disease in propensity score matched cohorts. Values are *n* (%) unless specified otherwise. (LS = lymphoscintigraphy, IQR = interquartile range, BMI = body mass index, ASA = American Society of Anesthesiologists, HR = hormone receptor, HER2 = human epidermal growth factor receptor 2, NS = not significant)

Total (1040)	pN0 (*n* = 799)	pN+ (*n* = 241)	Univariable analysis	Multivariable + odds ratio, 95% CI
LS			*p* < 0.0001	*p* < 0.0001
Successful	573 (71.7)	207 (85.9)		Reference
Failed, second injection administered	226 (28.3)	34 (14.1)		0.42 (0.28–0.64)
Age category (years)			*p* = 0.4430	
< 50	41 (5.1)	16 (6.6)		
50–69	536 (67.1)	152 (63.1)		
70+	222 (27.8)	73 (30.3)		
Surgery (breast)			*p* = 0.0023	NS*
Breast Conserving Surgery	750 (93.9)	212 (88.0)		
Mastectomy	49 (6.1)	29 (12.0)		
BMI categories (kg/m^2^)			*p* = 0.0092	NS*
15–20	17 (2.1)	10 (4.2)		
20–25	210 (26.3)	56 (23.2)		
25–30	302 (37.8)	70 (29.1)		
30–35	172 (21.5)	61 (25.3)		
35–40	74 (9.3)	29 (12.0)		
> 40	24 (3.0)	15 (6.2)		
ASA class			*p* = 0.2038	
ASA 1	90 (11.3)	33 (13.7)		
ASA 2	465 (58.4)	123 (51.0)		
ASA 3	231 (29.0)	80 (33.2)		
ASA 4	10 (1.3)	5 (2.1)		
Quadrant			*p* = 0.0721	
Lower lateral	102 (12.8)	29 (12.0)		
Lower medial	58 (7.3)	13 (5.4)		
Central	12 (1.5)	6 (2.5)		
Upper lateral	410 (51.3)	145 (60.2)		
Upper medial	217 (27.2)	48 (19.9)		
Cancer type			*p* = 0.7000	
Ductal	673 (84.2)	208 (86.3)		
Lobular	88 (11.0)	24 (10.0)		
Other	38 (4.8)	9 (3.7)		
TNM-T stage			*p* < 0.0001	*p* < 0.0001
T1a	34 (4.3)	3 (1.2)		Reference
T1b	245 (30.7)	31 (12.9)		1.10 (0.31–3.90)
T1c	370 (46.3)	107 (44.4)		2.52 (0.74–8.60)
T2	144 (18.0)	93 (38.6)		5.25 (1.51–18.22)
T3	6 (0.8)	7 (2.9)		18.49 (2.90-117.80)
Multifocality			*p* = 0.0505	NS*
No	676 (84.6)	191 (79.3)		
Yes	123 (15.4)	50 (20.7)		
HR			*p* = 0.3107	
positive	740 (93.2)	229 (95.0)		
negative	54 (6.8)	12 (5.0)		
HER2			*p* = 0.6087	
positive	58 (7.3)	20 (8.3)		
negative	736 (92.7)	221 (91.7)		
Proliferation (%). mean (IQR)	14 (7–25)	15 (10–25)	*p* = 0.0316	NS*
Grade			*p* = 0.0002	*p* = 0.0066
1	202 (27.0)	32 (14.0)		Reference
2	380 (50.8)	145 (63.6)		1.79 (1.15–2.77)
3	166 (22.2)	51 (22.4)		1.14 (0.67–1.95)

## Discussion

This study demonstrated that although SLNB is technically equally successful after the first injection and after a second injection following a failed initial attempt, metastases are significantly less frequently detected in the latter group (OR 0.42, *p* < 0.0001).

Historically, it has been reported that failure of LS may be associated with a higher nodal stage [16]. However, recently published studies suggest that no such association exists [17]. In the present study, the nodal stage was lower among the patients with failed LS. In multivariable analysis, a repeat injection yielded a similar number of lymph nodes as the first injection, but the probability of detecting SLNB metastasis was approximately half (OR 0.42) of that observed after the first injection.

It is important to note that early studies on SLNB were conducted at a time when axillary imaging was less accurate than it is today. A plausible explanation is that in cases where lymphatic obstruction previously prevented successful LS, modern imaging modalities are now able to identify patients with a high nodal burden more effectively. Consequently, the clinical significance of failed LS may have changed. As shown in the present study, a second subareolar injection after failed LS led to an equivalent SLNB identification rate, but fewer axillary metastases. Thus, the success of primary LS correlated with the presence of axillary metastases. One possible explanation for this finding is that SLN non-visualization in the contemporary clinical setting may reflect alternative lymphatic drainage rather than technical failure. This interpretation is supported, in part, by the observation that the difference was most pronounced in medial tumors, which are known to be associated with parasternal lymphatic drainage. Further prospective studies are required to validate this hypothesis [18, 19].

Notably, tumors located in the upper medial quadrant were more frequently associated with non-visualizing in LS (26.2% vs. 18.1%) whereas centrally located tumors were relatively underrepresented in the group (1.5% vs. 4.4%). Medial quadrants have also previously been found to be an influencing factor in failed LS [20]. Some studies show that tumor location does not significantly influence the success of LS, but these studies also note that the small sample size may affect the results of the study [21, 22]. These findings apply to patients without a clinically notable axillary metastasis burden (cN0-patients), which would have been detected during the preoperative axillary ultrasound triage, and may not be generalizable to settings without systematic axillary imaging or needle biopsy of suspicious lymph nodes, as preoperative exclusion of high-burden patients may influence the lower nodal positivity observed in the failed LS group. This topic warrants further investigation in contemporary clinical settings.

Our findings have two distinct implications. First, they support the use of reinjection as a pragmatic strategy that may restore the technical feasibility of SLNB after failed initial LS. Second, patients managed after failed LS demonstrated different nodal characteristics compared with those with initially successful mapping. Because of the retrospective nature of this study and the non-standardized reinjection protocol, these observations should be considered hypothesis-generating and should not be interpreted as evidence of an underlying biological mechanism.

In the present study, factors associated with the failure of the initial LS were older age, absence of multifocality, and medial tumor location. These factors remained independently associated with failed LS after a multivariable analysis.

Among our findings certain patient-related factors, particularly BMI, play a significant role in LS outcomes. High BMI has previously been associated with reduced imaging sensitivity [16], likely due to attenuation of gamma radiation from the radioisotope and altered lymphatic flow path dynamics [20, 23]. The observed association between a high BMI-category and failed LS in our findings supports the concept and highlights the importance of considering patient body composition when interpreting imaging results. Age was also identified as a significant factor in regard to the possibility of failure in LS. Aging has been documented to cause alterations in lymphatic function, including reduced flow and structural changes in lymph nodes which may contribute to decreased tracer migration and visualization [24, 25].

### Limitations

This study has several limitations. First, its retrospective design limits the ability to infer causality and introduces potential selection bias. Second, the use of pre-existing data may result in missing or inaccurate information. Additionally, not all relevant confounding variables could be controlled for, leaving the possibility of residual confounding. The study population was derived from a single center, which may limit the generalizability of the findings. Finally, changes in clinical practices over time may have influenced the observed outcomes.

A further limitation is that the second injection was performed only in patients with failed initial LS. The interpretation of LS reflected routine clinical practice, and the distinction between absent and equivocal uptake may involve some degree of subjective judgement. The decision to proceed with reinjection was based on institutional practice and involved practical considerations related to the planned surgery rather than a strictly standardized protocol. Consequently, the exposure was not randomized, and the study reflects a real-world institutional practice pathway rather than a controlled intervention. This may have resulted in a small degree of misclassification within the single-injection group. However, because this subgroup represented only approximately 5–8% of the single-injection population, any resulting residual bias is unlikely to have materially influenced the statistical analyses or altered the overall conclusions of the study. Although propensity score matching improved balance in measured covariates, it cannot address unmeasured factors such as injection depth, tracer dose, radiologist-specific practice, tumor palpability, imaging timing, individual lymphatic anatomy, or subtle clinical selection for reinjection, and residual confounding from these unmeasured factors cannot be excluded. Consequently, the study design does not allow conclusions regarding the causal effect of the second injection itself, and the results should be interpreted as demonstrating associations between LS failure and surgical outcomes rather than proving superiority or inferiority of either injection strategy.

Finally, follow-up data regarding axillary recurrence and false-negative SLN biopsy rates were not available. Therefore, the present study was limited to evaluating technical success and pathological nodal status at the time of surgery, and no conclusions can be drawn regarding the long-term oncologic implications of reinjection after failed LS.

## Conclusions

Second subareolar radiotracer injection after failed initial LS appears to be an effective salvage strategy for maintaining SLNB technical success, with SLN detection rates equivalent to those achieved after an initially successful injection. The observed lower nodal positivity rate among patients with failed initial LS may reflect patient selection through contemporary preoperative axillary ultrasound, altered lymphatic drainage patterns or other biological differences, and should be interpreted as hypothesis-generating. Further studies are required to clarify the clinical significance of these findings.

## Data Availability

The data used in this study contain sensitive personal health information and cannot be shared publicly. Access to the data is subject to permission from the relevant data controller and/or the Finnish data permit authority in accordance with the Finnish Act on the Secondary Use of Health and Social Data (552/2019).
